# Awareness of Opioid-Related Adverse Effects and Opioid-Sparing Preferences Among Medical Students and Interns: A Cross-Sectional Study

**DOI:** 10.7759/cureus.106428

**Published:** 2026-04-04

**Authors:** Yahia Atef Albashtawi, Sama A Shadid, Celeen Al Dabbas, Mera M Damiri, Amal I Alawadat, Layan Alamir, Zainab S Satea, Nizar Habash, Bassel F Qiqieh, Sumaya H Tamimi

**Affiliations:** 1 Urology, Abdali Hospital, Amman, JOR; 2 Pharmacy, Jordan University of Science and Technology, Irbid, JOR; 3 Faculty of Medicine, University of Jordan, Amman, JOR; 4 Faculty of Medicine, Al-Balqa Applied University, As-Salt, JOR; 5 General Practice, Jordan University Hospital, Amman, JOR; 6 Faculty of Medicine, Yarmouk University, Irbid, JOR; 7 Faculty of Medicine, Jordan University of Science and Technology, Irbid, JOR

**Keywords:** adverse effects, awareness, jordan, medical education, opioids, opioid-sparing analgesia, prescribing patterns

## Abstract

Background

Opioid-related adverse effects and inappropriate opioid prescribing remain major contributors to preventable morbidity. Medical education plays a crucial role in shaping early prescribing behaviours, particularly among future healthcare professionals. However, data on opioid-related awareness and opioid-sparing preferences among medical students and interns in Middle Eastern settings remains limited.

Objectives

This study aims to evaluate the level of awareness of opioid-related adverse effects and attitudes toward opioid‑sparing strategies among medical students and interns in Jordan. A secondary objective was to examine which aspects of medical training were associated with higher knowledge levels.

Methods

We conducted a cross-sectional, questionnaire-based study among medical students and interns (n = 250) in Jordan. Data were collected between September 2025 and November 2025 using an anonymous, self-administered online survey distributed via social media platforms and university email lists. A convenience sampling approach was used, supplemented by snowball sampling through medical student networks. The survey assessed demographic characteristics, prior pain management education, knowledge of opioid-related adverse effects, and attitudes toward opioid-sparing strategies. Knowledge was measured using four multiple-choice items and summarized as a total knowledge score (0-4). High knowledge was defined a priori as a score of ≥75% correct responses (3 or 4 out of 4). Attitudes were assessed using Likert-scale items (range 4-20). Descriptive statistics were calculated, and associations between participant characteristics and high knowledge level were examined using chi-square tests.

Results

The mean knowledge score was 2.68 ± 0.90, with 19.2% of participants classified as having high knowledge. Correct response rates across individual knowledge items ranged from 58.0% to 74.0%. The mean attitude score toward opioid-sparing approaches was 14.98 ± 1.94, indicating generally favourable attitudes. No significant associations were observed between high knowledge level and training stage (χ²(2) = 0.263, p = 0.877), completion of surgical rotation (χ²(1) = 1.549, p = 0.213), or prior pain education (χ²(2) = 1.079, p = 0.583).

Conclusion

Medical students and interns demonstrated moderate knowledge of opioid-related adverse effects alongside generally positive attitudes toward opioid-sparing strategies. However, higher knowledge was not associated with the training stage or prior educational or clinical exposure. These findings suggest a potential need for more structured and clinically integrated opioid education early within undergraduate medical curricula.

## Introduction

Prescribing opioids for acute and chronic pain remains a common clinical practice, despite growing evidence linking their use to adverse outcomes, including respiratory depression, dependency, and overdose [1,2]. While medical communities have increasingly prioritized opioid stewardship and opioid-sparing strategies, inappropriate prescribing continues to pose a significant and avoidable threat to opioid-naïve patients [3].

Medical education is a foundational stage where a doctor's prescribing habits and professional attitudes are first established. Studies from North America and Europe suggest that medical students report feeling underprepared to implement opioid-sparing strategies or identify patients at high risk for opioid-related adverse events [4]. Unaddressed knowledge gaps during medical school can frequently result in unsafe prescribing behaviors during early clinical practice [5].

Most opioid education research originates from a Western context, thus creating a large evidence gap for Middle Eastern and lower-income regions, with significant differences in prescribing environments, clinical exposures, and curricula. Understanding baseline knowledge and attitudes among medical students and interns will be important in guiding targeted educational interventions ahead of their transition to independent prescribing practices. The aim of the study was to assess awareness of opioid-related adverse effects and attitudes toward opioid-sparing strategies among medical students and interns in Jordan, and to examine which aspects of medical training were associated with higher knowledge levels.

## Materials and methods

Study design and setting

We conducted a cross-sectional survey among medical students and interns in Jordan. Data were collected between September 2025 and November 2025 using an anonymous, self-administered online questionnaire distributed via social media platforms (Facebook and WhatsApp (Meta Platforms, Inc., Menlo Park, California, United States) groups for medical students) and official university email lists. Participation was voluntary and anonymous.

Study population

Eligible participants included undergraduate medical students in pre-clinical and clinical years, as well as medical interns enrolled in any of the six public and private medical schools in Jordan (located in Amman, Irbid, and other major cities). All incomplete survey responses and responses from participants who did not meet the inclusion criteria were excluded. No incentives were offered for participation.

A sample size of 250 participants was determined based on feasibility and an expected prevalence of high knowledge of approximately 20%, derived from pilot data. For a 95% confidence level and a 5% margin of error, the minimum required sample size was calculated as approximately 246 participants. A convenience sampling strategy was employed, supplemented by snowball sampling through medical student networks and social media groups to reach participants across different training levels and universities. While this approach does not guarantee a fully representative sample, it was deemed appropriate for this exploratory cross-sectional study.

Survey instrument

The questionnaire was developed by the authors to assess opioid-related knowledge and attitudes in a concise format appropriate for medical trainees. It included sections addressing demographic characteristics, training level, prior exposure to pain management education, knowledge of opioid-related adverse effects, and attitudes toward opioid-sparing strategies. The full questionnaire is provided in the Appendices.

The survey was piloted on 20 medical students to assess clarity, comprehensibility, and completion time. Based on pilot feedback, minor wording adjustments were made to improve item clarity. Pilot responses were not included in the final analysis. No formal reliability testing (e.g., Cronbach's alpha) was conducted, which is acknowledged as a limitation.

Knowledge was assessed using four multiple-choice questions covering recognition of common opioid adverse effects, identification of respiratory depression risk, recognition of increased risk among opioid‑naïve patients, and identification of a non-opioid adverse effect. Each correct response was awarded one point, generating a total knowledge score ranging from 0 to 4. High knowledge was defined a priori as a score of three or four (≥75% correct responses), reflecting adequate recognition of core adverse-effect domains.

Attitudes toward opioid-sparing strategies were assessed using four Likert-scale statements addressing prioritisation of non-opioid analgesics, minimising opioid exposure when clinically appropriate, and general stewardship perspectives. Responses were scored on a five-point scale and summed to create a composite attitude score ranging from 4 to 20, with higher scores indicating more favourable attitudes.

Statistical analysis

Statistical analyses were conducted using JASP software version 0.95.4 (JASP Team, University of Amsterdam, Amsterdam, Netherlands). Categorical variables were summarised as frequencies and percentages, while continuous variables were summarised as means and standard deviations. Knowledge and attitude scores were described using measures of central tendency and dispersion. Associations between high knowledge level and training-related variables were examined using chi-square tests. A p-value < 0.05 was considered statistically significant.

Ethical considerations

The study involved an anonymous, minimal-risk electronic survey. No identifying personal information was collected. Participation was voluntary, and electronic informed consent was obtained prior to survey completion. In accordance with national and institutional guidelines for minimal-risk, anonymous survey research involving adults, formal Institutional Review Board approval was not required.

## Results

A total of 250 medical students and interns completed the survey. Participants represented all stages of undergraduate medical training and internship, with most enrolled at public universities (73.6%). Among the participants, 64.0% reported having completed a surgical rotation, and 50.4% reported prior exposure to pain management education. A large portion of the participants (38.0%) were aged 21-23 years, closely followed by ages 18-20 years (28.0%). The majority were female (54.4%). Most participants were clinical students (43.6%) with only 20.0% being medical interns (Table 1).

**Table 1:** Characteristics of medical students and interns who completed the opioid awareness survey (N = 250)

Characteristic	Frequency (Percentage)
Age group (years)	
18–20	70 (28.0)
21–23	95 (38.0)
24–26	59 (23.6)
≥27	26 (10.4)
Gender	
Male	106 (42.4)
Female	136 (54.4)
Prefer not to say	8 (3.2)
Training level	
Pre-clinical student	91 (36.4)
Clinical student	109 (43.6)
Medical intern	50 (20.0)
University type	
Public	184 (73.6)
Private	66 (26.4)
Completed surgical rotation	
No	90 (36.0)
Yes	160 (64.0)
Prior pain management education	
No	86 (34.4)
Yes	126 (50.4)
Not sure	38 (15.2)

Correct response rates for individual knowledge items ranged from 58.0% to 74.0%, with the highest correct response observed for identification of non-opioid adverse effects (74.0%) and the lowest for identification of risk in opioid-naive patients (58.0%) (Table 2).

**Table 2:** Proportion of correct responses to opioid-related knowledge questions among medical students and interns

Knowledge item	Correct response, n (%)
Identification of common opioid adverse effects	169 (67.6)
Recognition of respiratory depression risk	172 (68.8)
Identification of higher risk in opioid-naïve patients	145 (58.0)
Correct identification of non-opioid side effect	185 (74.0)

The mean knowledge score was 2.68 ± 0.90, with a median of 3.0. A total of 48 participants (19.2%) met criteria for high knowledge. Attitudes toward opioid-sparing strategies were generally favourable. The mean attitude score was 14.98 ± 1.94, with a median score of 15.0 (Table 3). Most participants indicated agreement with statements prioritising non-opioid analgesics when clinically appropriate. 

**Table 3:** Distribution of attitude scores toward opioid-sparing strategies among medical students and interns

Score	Mean ± SD	Median	Range
Knowledge score (0–4)	2.68 ± 0.90	3.0	0–4
Attitude score (4–20)	14.98 ± 1.94	15.0	10–20

No statistically significant associations were observed between high knowledge level and training stage, completion of a surgical rotation, or prior pain management education (Table 4).

**Table 4:** Associations between participant characteristics and high opioid-related knowledge level

Variable	High knowledge, n (%)	χ² (df)	p-value
Level of training	Pre-clinical 19/91 (20.9)	0.263 (2)	0.877
	Clinical 20/109 (18.3)		
	Intern 9/50 (18.0)		
Completed surgical rotation	Yes 27/160 (16.9)	1.549 (1)	0.213
	No 21/90 (23.3)		
Prior pain management education	No 17/86 (19.8)	1.079 (2)	0.583
	Yes 26/126 (20.6)		
	Not sure 5/38 (13.2)		

## Discussion

This cross-sectional study evaluated awareness of opioid-related adverse effects and attitudes toward opioid-sparing strategies among medical students and interns in Jordan. Participants demonstrated moderate knowledge, with fewer than one-fifth achieving high knowledge. Notably, knowledge levels did not improve with advancing training stage or prior exposure to pain education, suggesting limited progression of opioid-related competencies across undergraduate medical training.

These findings are consistent with prior studies from North America and Europe reporting insufficient pain education and limited preparedness among medical trainees in opioid risk assessment [6-8]. Clinical exposure alone does not appear to ensure competency, likely reflecting fragmented or non-longitudinal integration of opioid-related content within medical curricula [9]. Importantly, recognition of increased risk among opioid-naïve patients showed the lowest correct response rate (58.0%), which is clinically significant given the heightened susceptibility of this group to respiratory depression and overdose [10]. Current guidelines emphasise the importance of structured opioid stewardship education to address such gaps early in training [11].

Despite these knowledge limitations, attitudes toward opioid-sparing strategies were generally favourable. This aligns with existing literature indicating that medical trainees are receptive to minimising opioid use when supported by clear clinical guidance [12]. However, favourable attitudes alone may not translate into safe prescribing practices without adequate knowledge and structured educational reinforcement.

To further contextualise these findings, a recent systematic review of opioid prescribing education reported that isolated didactic teaching, in the absence of clinical integration, has limited impact on long-term knowledge retention and prescribing behaviour [11]. Additionally, studies from comparable Middle Eastern settings suggest that local prescribing practices and educational exposure may differ from Western contexts, underscoring the need for region-specific educational strategies [12]. Collectively, these findings support the need for longitudinal, clinically integrated opioid education tailored to local healthcare environments.

This study has several strengths. It provides novel region-specific data from a Middle Eastern context, includes participants across multiple stages of training, and evaluates both knowledge and attitudes using a structured scoring approach. Furthermore, the relatively large sample size enhances the reliability of the findings.

Several limitations should be considered. First, the cross-sectional design precludes causal inference regarding the relationship between training and knowledge levels. Second, the use of convenience and snowball sampling may have introduced selection bias, potentially overrepresenting individuals with greater interest in pain management. Additionally, due to the online distribution of the survey, the exact response rate could not be determined. Third, the knowledge assessment tool comprised only four items and may not fully capture clinical competence or depth of understanding. Fourth, the survey instrument was not formally validated for reliability (e.g., Cronbach’s alpha was not assessed). Finally, reliance on self-reported prior education introduces the possibility of recall bias. These limitations are common in exploratory cross-sectional survey studies and should be considered when interpreting the findings.

## Conclusions

Medical students and interns demonstrated moderate awareness of opioid-related adverse effects and generally positive attitudes toward opioid-sparing strategies. However, higher knowledge levels were not associated and did not improve with training stage, clinical exposure, or prior pain education, suggesting gaps in current educational approaches. These findings support the necessity for more structured and longitudinal opioid-related education during medical training to better prepare future prescribers for safe pain management. Importantly, the study provides valuable baseline knowledge, laying the groundwork for future research designed to evaluate the impact of targeted educational interventions on prescribing competence.

## Appendices

Survey questionnaire

**Table 5:** Structured Survey Questionnaire

Section	Question	Response Options
A. Participant Characteristics	Age group	○ 18–20 ○ 21–23 ○ 24–26 ○ ≥27
	Gender	○ Male ○ Female ○ Prefer not to say
	Current level of training	○ Pre-clinical medical student ○ Clinical-year medical student ○ Medical intern
	University type	○ Public ○ Private
	Completed a surgical rotation	○ Yes ○ No
	Received formal teaching on pain management or opioids	○ Yes ○ No ○ Not sure
B. Knowledge of Opioid-Related Side Effects	Which of the following is a common opioid-related adverse effect?	○ Constipation ○ Hypertension ○ Hyperglycemia ○ Bradycardia
	Respiratory depression is most strongly associated with:	○ High opioid doses ○ Short-term NSAID use ○ Acetaminophen overdose ○ Local anesthetics
	Which patient group is at higher risk of opioid-related adverse effects?	○ Opioid-naïve patients ○ Patients on long-term antibiotics ○ Young healthy adults only ○ Patients using topical analgesics
	Which of the following is NOT a recognized opioid side effect?	○ Nausea ○ Sedation ○ Physical dependence ○ Nephrolithiasis
C. Awareness of Opioid-Sparing Strategies	Opioid-sparing strategies aim to:	○ Reduce or avoid opioid use while controlling pain ○ Replace surgery with opioids ○ Increase opioid doses safely ○ Use opioids as first-line therapy
	Which is an opioid-sparing approach?	○ Multimodal analgesia ○ Opioid monotherapy ○ Increasing opioid duration ○ Avoiding non-opioid analgesics
	Which medication can be used as part of opioid-sparing pain control?	○ NSAIDs ○ Antihistamines ○ Antibiotics ○ Antipsychotics
D. Attitudes Toward Opioid Use	Opioids are overused in postoperative pain management.	○ Strongly disagree ○ Disagree ○ Neutral ○ Agree ○ Strongly agree
	I feel confident identifying opioid-related side effects.	○ Strongly disagree ○ Disagree ○ Neutral ○ Agree ○ Strongly agree
	Non-opioid options should be considered before prescribing opioids.	○ Strongly disagree ○ Disagree ○ Neutral ○ Agree ○ Strongly agree
	Medical students and interns need more formal education on opioid safety.	○ Strongly disagree ○ Disagree ○ Neutral ○ Agree ○ Strongly agree
E. Preferences and Intended Practice	If you were involved in postoperative pain management, which would you prefer?	○ Opioid-first approach ○ Opioid-sparing approach ○ Depends on the clinical case
	Would you prefer to receive more training on opioid-sparing strategies?	○ Yes ○ No ○ Not sure
	Preferred method of learning	□ Lectures □ Workshops □ Online modules □ Clinical case discussions
F. Consent	I confirm that I am ≥18 years old and agree to participate voluntarily.	○ I agree to participate
